# Supplementary material for: Identification of photoperiod-induced specific miRNAs in the adrenal glands of Sunite sheep (Ovis aries)
Source: Front Vet Sci. 2022 Jul 22;9:888207. doi: 10.3389/fvets.2022.888207 (PMC9354845; doi:10.3389/fvets.2022.888207)
Supplement: Supplementary file 1 [file Data_Sheet_1.ZIP › Supplementary file/miRDeep2 file.zip!/miRDeep2 file/Know_Structure/LP42A2/oar-mir-3957.pdf]

miRBase precursor : oar-mir-3957
Total read count : 90
oar-miR-3957-5p read count : 69
oar-miR-3957-3p read count : 21
remaining reads : 0

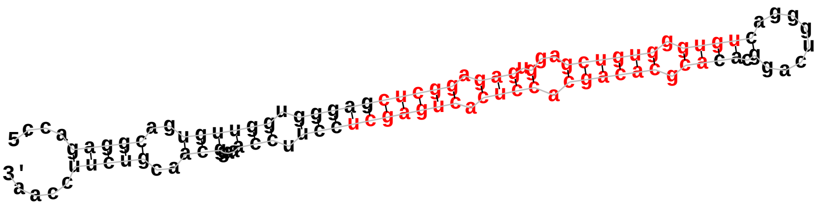

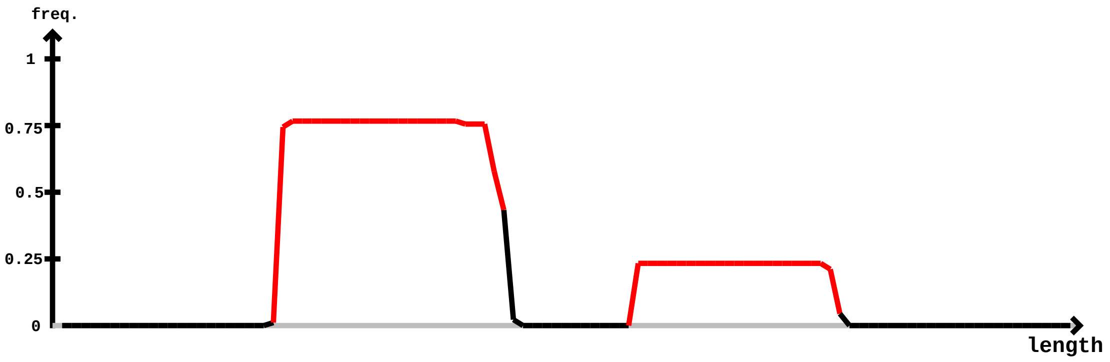

oar-miR-3957-5p

oar-miR-3957-3p

```
5'- ccagaggcaguguuggugggagcucggagaguggagcugugggugucagggucaggcacacgcacagcaccucacugagcuccuuccaaaggcaacgucuuccaa   -3'   exp
    ...(((((..((((((.((((((((((.(((.(..((((((.(((((........).)))).)))))).)))).))))))))))).)))...)))..)))))....   reads   mm   sample
    .....................gcucggagaguggagcugugggugu...........................................................   1       0    seq
    ......................cucggagaguggagcugug................................................................   1       0    seq
    ......................cucggagaguggagcugugggu.............................................................   15      0    seq
    ......................cucggagaguggagcugugggug............................................................   13      0    seq
    ......................cucggagaguggagcugugggugu...........................................................   35      0    seq
    ......................cucggagaguggagcugugggugucl..........................................................   2       0    seq
    .......................ucggagaguggagcugugggu.............................................................   1       0    seq
    .......................ucggagaguggagcugugggugu...........................................................   1       0    seq
    ...........................................................acgcacagcaccucacugag..........................   2       0    seq
    ...........................................................acgcacagcaccucacugagc.........................   15      0    seq
    ...........................................................acgcacagcaccucacugagcu........................   4       0    seq
```
